# The Cosmos Collaborative: A Vendor-Facilitated Electronic Health Record Data Aggregation Platform

**DOI:** 10.1055/s-0041-1731004

**Published:** 2021-01

**Authors:** Yasir Tarabichi, Adam Frees, Steven Honeywell, Courtney Huang, Andrew M. Naidech, Jason H. Moore, David C. Kaelber

**Affiliations:** 1 Center for Clinical Informatics Research and Education, The MetroHealth System, Cleveland, Ohio, United States; 2 Division of Pulmonary and Critical Care Medicine, Department of Internal Medicine, The MetroHealth System, Cleveland, Ohio, United States; 3 School of Medicine, Case Western Reserve University, Cleveland, Ohio, United States; 4 Epic, Verona, Wisconsin, United States; 5 Department of Neurology, Northwestern University. Chicago, Illinois, United States; 6 Institute for Biomedical Informatics, Perelman School of Medicine, University of Pennsylvania, Philadelphia, Pennsylvania, United States; 7 Departments of Internal Medicine, Pediatrics, and Population and Quantitative Health Sciences, School of Medicine, Case Western Reserve University, Cleveland, Ohio, United States

**Keywords:** electronic health record, data aggregation, research network, health information exchange, collaboration

## Abstract

**Objective:**

Learning healthcare systems use routinely collected data to generate new evidence that informs future practice. While implementing an electronic health record (EHR) system can facilitate this goal for individual institutions, meaningfully aggregating data from multiple institutions can be more empowering. Cosmos is a cross-institution, single EHR vendor-facilitated data aggregation tool. This work aims to describe the initiative and illustrate its potential utility through several use cases.

**Methods:**

Cosmos is designed to scale rapidly by leveraging preexisting agreements, clinical health information exchange networks, and data standards. Data are stored centrally as a limited dataset, but the customer facing query tool limits results to prevent patient reidentification.

**Results:**

In 2 years, Cosmos grew to contain EHR data of more than 60 million patients. We present practical examples illustrating how Cosmos could further efforts in chronic disease surveillance (asthma and obesity), syndromic surveillance (seasonal influenza and the 2019 novel coronavirus), immunization adherence and adverse event reporting (human papilloma virus and measles, mumps, rubella, and varicella vaccination), and health services research (antibiotic usage for upper respiratory infection).

**Discussion:**

A low barrier of entry for Cosmos allows for the rapid accumulation of multi-institutional and mostly de-duplicated EHR data to power research and quality improvement queries characteristic of learning healthcare systems. Limitations are being vendor-specific, an “all or none” contribution model, and the lack of control over queries run on an institution’s healthcare data.

**Conclusion:**

Cosmos provides a model for within-vendor data standardization and aggregation and a steppingstone for broader intervendor interoperability.

## Introduction

Facilitated by the introduction of the Health Information Technology for Economic and Clinical Health Act of 2009, electronic health records (EHRs) have become ubiquitous across the United States.^1,2^ In digitizing paper records and processes, healthcare systems gained the potential for immediate access to the data that they needed to analyze and refine their practices and improve their outcomes. This step is arguably an essential one for the development of a true “learning healthcare system.”^3,4^

A natural extension of the learning healthcare system framework involves leveraging the collective experiences of numerous healthcare systems through multisite collaborations. This is the driving principle behind several successful quality initiatives, such as the American College of Surgeon’s National Surgical Quality Improvement Program (NSQIP).^5–7^ Unfortunately, EHR data tend to be siloed within institutions, and efforts for wider intersystem aggregation are often hampered by regulatory, technical, and financial barriers.^8^ Despite these limitations, many initiatives have been successful, including several national research collaboratives,^9–11^ surveillance networks,^12^ and regional public health initiatives.^13,14^

Tarabichi et al recently described a federated, vendor-facilitated (Epic, Verona, Wisconsin, United States) EHR data aggregation initiative known as the Aggregate Data Program (ADP).^15^ The ADP was a proof-of-concept disease-specific registry that reduced the barrier of entry to collaborators by leveraging native EHR tools for the periodic submission of aggregated EHR data to a central repository. The success of that initiative laid the groundwork for Cosmos. Like the ADP, Cosmos is vendor-facilitated with robust customer input into its design and implementation. Cosmos goes further by leveraging standard health information exchange infrastructures to continuously and automatically retrieve, harmonize, and collate a greater variety of discrete data points from participating organizations. In addition, Cosmos empowers its contributors with a web-based query building interface that allows users to go beyond a priori determined questions available in the ADP. Here, we describe Cosmos as it exists at the time of publication and provide examples for how the data and platform may be used to further public health surveillance, quality improvement, and research initiatives. This manuscript is intended to be the first formal description of this initiative, and these use cases were selected to demonstrate Cosmos’ structure, functions, and capabilities.

## Methods

### Program Governance and Structure

Cosmos is managed by the Epic corporation (Verona, Wisconsin, United States), with guidance from elected representatives of their customer community (the Governing Council). The council currently has 11 members, consisting of executives, researchers, and clinicians from the organizations that participate in Cosmos. Council terms are 3 years, and members cannot serve consecutive terms but can be renominated after one election cycle. The council is responsible for promoting best practices and advising the vendor on the direction of the collaboration.

Cosmos is an opt-in service for Epic EHR customers. To participate, organizations must agree to the Cosmos guidelines (called the Rules of the Road). These guidelines are codeveloped by the Epic corporation and the Governing Council, and enforcement is ensured by both entities. The guidelines are not publicly published but are made available to all users of the Epic EHR platform.

### Data Structure and Quality Control

Cosmos contains a variety of data points per patient, spanning many discrete data variable types (Table 1). While Cosmos does use Epic’s own proprietary data model to store the data, it favors direct linkage to standardized ontologies over custom ones, relying mostly on Uniform Medical Language System data models such as Systematized Nomenclature of Medicine-Clinical Terms (SNOMED-CT), Logical Observation Identifiers Names and Codes (LOINC), RxNorm (standardized nomenclature for drugs in the United States), and CVX (vaccine administration codes).^16^ Other data models leveraged include the National Uniform Claim Committee health care provider taxonomy,^17^ National Uniform Billing Committee discharge codes (NUBC FL 17),^18^ and International Classification of Disease revisions 9 and 10.^19^ The majority of data transmitted to Cosmos must conform to the aforementioned ontologies before transmission. In rare circumstances, Cosmos curates additional nonstandard data when the common standards are deemed insufficient, such as in the case of documentation of birth control classification methodologies not well characterized in SNOMED-CT.

For most items, Cosmos uses the same mapping process that Epic customers need to complete for standard clinical health information exchange. Most institutions will have adopted their native data models to reflect and/or link to standard ontologies at the outset, but some additional mapping or manual corrections may need to be addressed by individual sites before data submission. A small subset of nonstandardized data types, such as race, ethnicity, and reason for visit, are mapped by the vendor.

Once an institution agrees to contribute to Cosmos, the vendor provides a feedback loop between Cosmos and its contributors through periodic data quality reports. The reports include metrics on mapping completeness, identifying most frequently received unmapped values, as well as potential data irregularities, including date outliers, laboratory results with missing units or the reception of low rates of documentation of an important birth metric. Data completeness for important variables is considered and scored, dependent on the variable type. Anomalies in the frequency of data variables are monitored in longitudinal fashion, with attention to large relative changes in count data. Such changes are flagged and prompt manual review to determine if they are expected (such as increases in influenza vaccination rates in the fall). The vendor also assesses laboratory data distributions to detect potentially incorrect LOINC mapping.

### Data Submissions and Triggers

The data submitted to Cosmos can be divided into two broad categories: “backload” data and “triggered” data. Backload data consists of records that existed prior to an organization’s involvement in Cosmos. Triggered data are prospectively accumulated and submitted to Cosmos based on event-driven triggers, such as encounter closure, a result being filed, or a chart being corrected. Encounter record submissions are triggered by 7 days of inaction, even if the encounter has not been closed. This prevents the delay of transmission of discrete objective data, such as completed laboratory results, due to incomplete documentation which would not otherwise be transmitted to Cosmos.

**Fig. 1:**
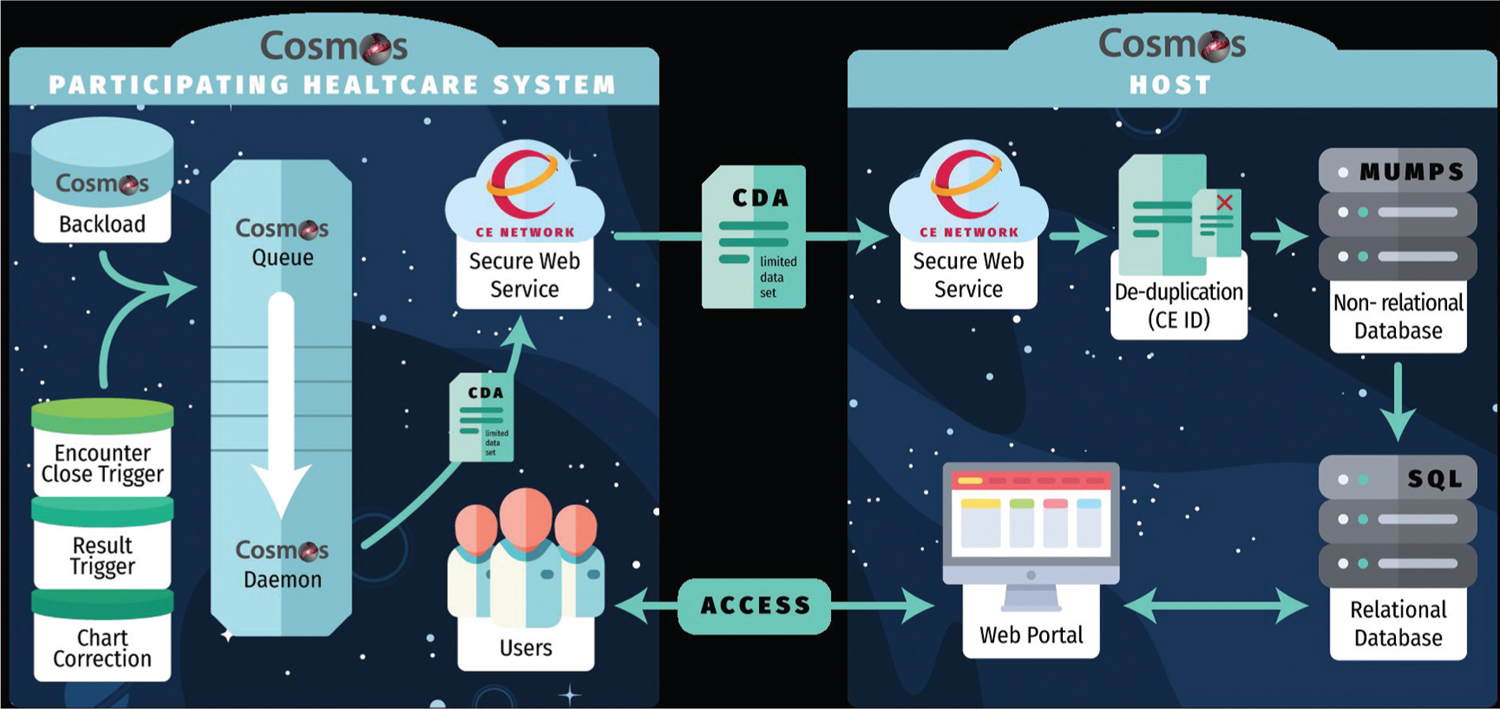
Schematic for the Cosmos architecture. Backload and triggered data move onto the Cosmos queue, where it is processed by the Cosmos daemon. Data are transmitted to the Cosmos host as encrypted HL7 C-CDA documents over the Care Everywhere Network. Patient deduplication is performed by using a hashed copy of a Care Everywhere ID, after which data are filed in a Massachusetts General Hospital Utility Multi-Programming System nonrelational database, and then a search query language relational database. All participating healthcare systems communicate with the same Cosmos host. Users can access the data via a web portal.

Backload and triggered data are prioritized for submission via the Cosmos Queue (Fig. 1). Priority is given to more recent events. To alleviate computational strain on both the submitting organization and on Cosmos, the backload is often performed in stages, limiting submissions to the most recent few years. Background processes advance through the queue and prepare each record for transmission to Cosmos.

### Data Privacy and Transmission

Patient privacy and data security are core concerns for the EHR vendor and all contributing customers. Data are transmitted to Cosmos through encrypted health level 7 Consolidated-Clinical Document Architecture (C-CDA) documents over a secure existing clinical health information exchange platform (the Care Everywhere network). Care Everywhere is a “point-to-point” or nonfederated peer-to-peer network health information exchange mechanism, which has been used since 2008 to transmit hundreds of millions of patients’ charts between organizations that use the Epic EHR for clinical care purposes (Fig. 1).^20^

Cosmos contains a limited dataset, as defined by the Health Insurance Portability and Accountability Act of 1996.^21^ The initiative has been designed with safeguards to prevent submission of protected health information, with exceptions including dates of birth, dates of service/testing, 5 digit zip code, and a unique internal identifier (Care Everywhere [CE] ID). The CE ID is used to identify the same patient across multiple EHR instances and does not encompass any patient information.^22^ Before being transmitted to Cosmos, the CE ID is hashed via the SHA-256 cryptographic function so that it cannot be used to reveal a patient’s identity.^23^ Studies have shown that the CE ID correctly identifies the same patient in different EHRs at least approximately 85% of the time.^22,24^ A more recent study in Los Angeles revealed no false positives and an estimated false negative rate of close to 3%.^25^ By leveraging the CE ID, most information about the same patient in multiple healthcare systems contributing to Cosmos is combined into a single patient record. This reduces double counting, and the provides a more temporally complete patient record when care is fractured between different institutions.

Free text data from notes or comments are not submitted to Cosmos. Free text data from laboratory results, however, are submitted after passing a strict inclusion list filter.

### Data Access

Individuals from healthcare systems contributing data to Cosmos can query data in Cosmos through a secure web application. The web application leverages a graphical user interface that allows users to build modular queries without writing any code. Queries return cohort counts or summary measures (such as minimum, maximum, mean, or standard deviations of included continuous variables). Users can use Boolean logic to combine their criteria and place relative temporal restrictions on queries. For example, a query could retrieve the average age and the numberof patients who receiveda certainvaccine and receiveda follow-up booster within 12 months. Because Cosmos only returns population level data and obscures counts <11 patients (only reported as “10 or fewer”), data returned from Cosmos queries do not constitute human subjects research. As a result, end-users do not require institutional review board approval for research purposes.

### Current State of the Registry

**Fig. 2:**
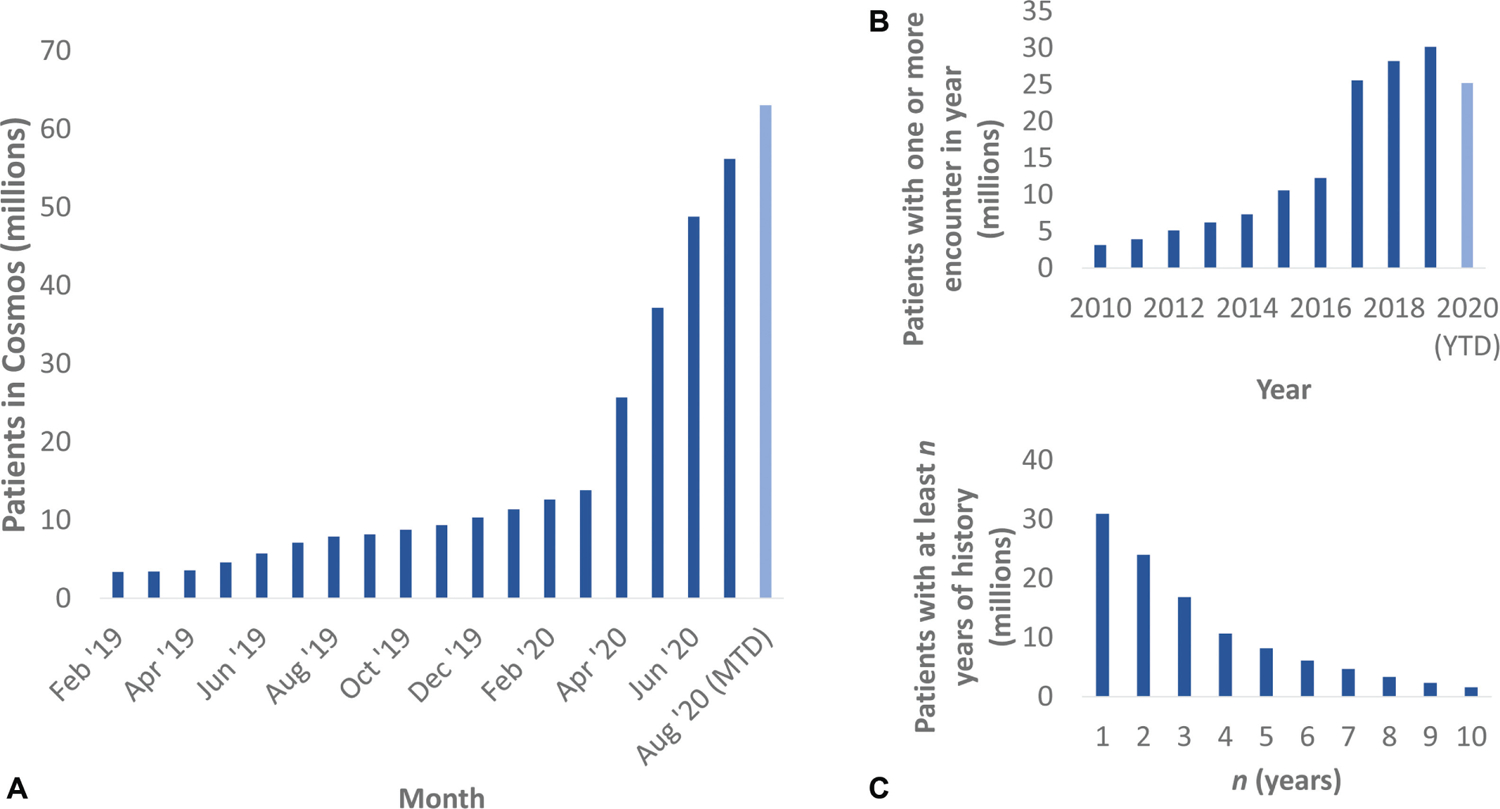
Cosmos characteristics as of August 2020. (A) Cumulative number of unique patients in Cosmos as a function of time. (B) Number of unique patients with an encounter in Cosmos by year. (C) Length of time between first and latest encounter in Cosmos per unique patient. To generate this query, available laboratory results that included “influenza” or “severe acute respiratory syndrome” in their titles were screened to determine a rapid diagnostic test, as opposed to an antibody study (the resulting Logical Observation Identifiers Names and Codes are noted in the supplementary material). MTD, month to date; YTD, year to date.

Cosmos has been accepting data since 2018, with historical (backloaded) data extending as far back as 2005. As of August 2020, Cosmos has data from more than 60 million unique patients (Fig. 2A), with representation from all 50 states. These contributions come from 75 participating sites (25 academic medical centers, 50 nonacademic medical centers, and 5 children’s hospitals).

The backload submissions for many contributing organizations are ongoing, and therefore, most of the records in Cosmos reflect recent information. Despite this, for each year in the past decade Cosmos contains encounter records from that year for millions of patients (Fig. 2B). Additionally, over 15 million patients have at least 3 years of medical history in Cosmos, and over 1 million have at least 10 years (Fig. 2C).

## Statistical Approach to Sample Use Cases

Raw count data for the use cases were obtained directly from the native Cosmos query interface. Measures of prevalence are limited by EHR documentation completion. Descriptive statistics were provided for most measures and comparisons to alternative data sources were qualitative. Confidence intervals were calculated by using the binomial exact method, and proportions compared with Chi-square testing where applicable. All analyses were conducted in R (version 3.5.1) and figures generated with ggplot2.^26,27^

No institutional review board approval was needed due to the aggregate de-identified nature of the data that was accessed. This publication and its contents were approved by the Cosmos Governing Council.

## Results

### Chronic Disease Surveillance: Asthma and Obesity

**Fig. 3:**
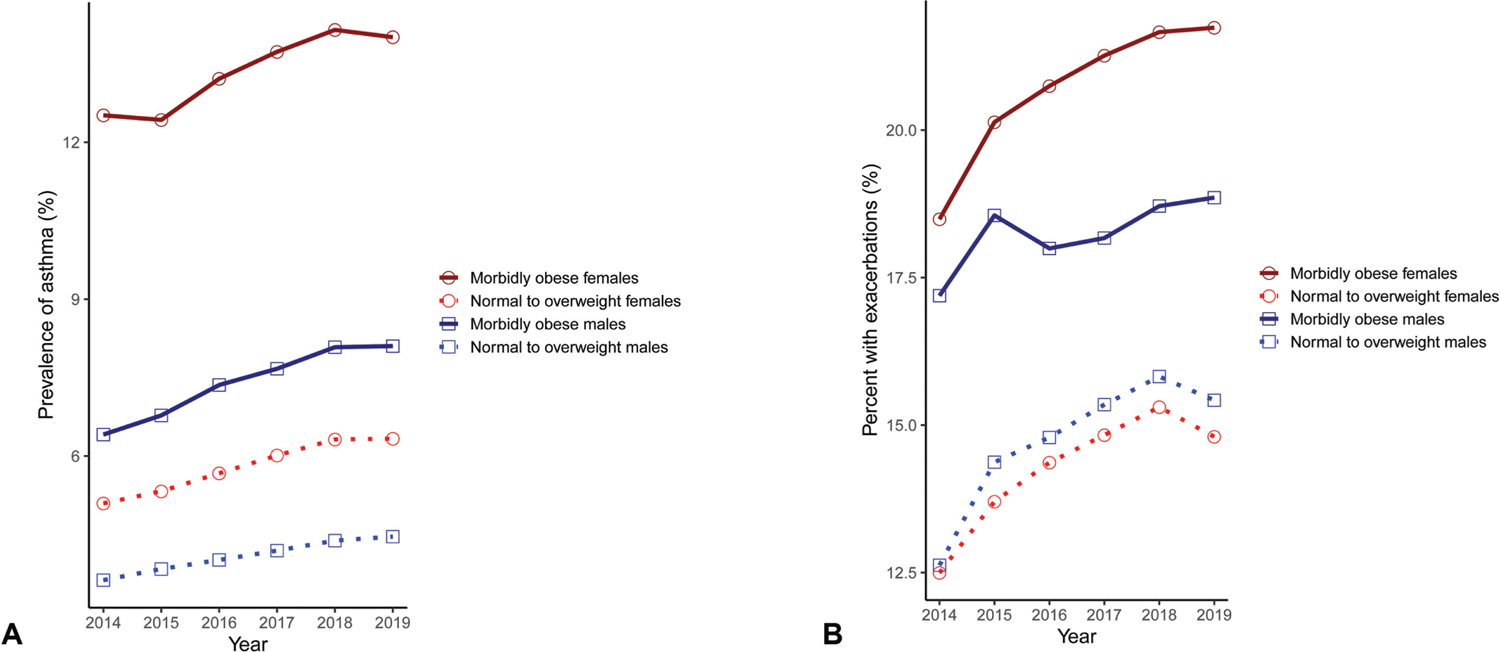
Relationships between asthma and obesity in Cosmos. (A) The prevalence of asthma within different obesity classes, stratified by sex. (B) The percentage of asthmatics who have at least one encounter with a SNOMED diagnosis of asthma exacerbation, stratified by sex, and weight class. Annual asthma prevalence was defined as an encounter or problem list diagnosis during that year that mapped to a SNOMED diagnosis of asthma (SNOMED-CT 195967001). Asthma exacerbations were indicated by the presence of an encounter or problem list diagnosis that mapped to the SNOMED “exacerbation of asthma” concept (SNOMED-CT 281239006). Normal to overweight was defined as a BMI <30 kg/m^2^, obese as a BMI of 30 to <40 kg/m^2^, and morbidly obese as a BMI ≥40 kg/m^2^ during each calendar year. BMI, body mass index; SNOMED-CT, Systematized Nomenclature of Medicine-Clinical Term.

One of the major functions of the Centers for Disease Control (CDC) is to measure and monitor important public health trends. The intersection of asthma and obesity, for instance, is of recent interest.^28^ Cosmos enables combining elements of administrative data, vital signs, and demographics to study EHR asthma prevalence, and the likelihood of a clinically noted exacerbation along strata of sex and body mass index (BMI) (Fig. 3). The ability to query vital sign data such as BMI makes the latter assessment more reliable than relying on diagnosis data alone.^29,30^ In our analysis for the year of 2019, the prevalence of asthma was significantly greater in morbidly obese woman than morbidly obese men (14.0 vs. 8.1%, *p* < 0.001), consistent with recent data from the CDC.^28^ In addition, our data show that morbidly obese asthmatic women were significantly more likely to experience clinically significant exacerbations compared with morbidly obese asthmatic men (21.7 vs. 18.9%, *p* < 0.001), mirroring evidence from a growing literature describing this phenomenon.^31,32^

### Syndromic Surveillance: Seasonal Influenza and the Novel Coronavirus

**Fig. 4:**
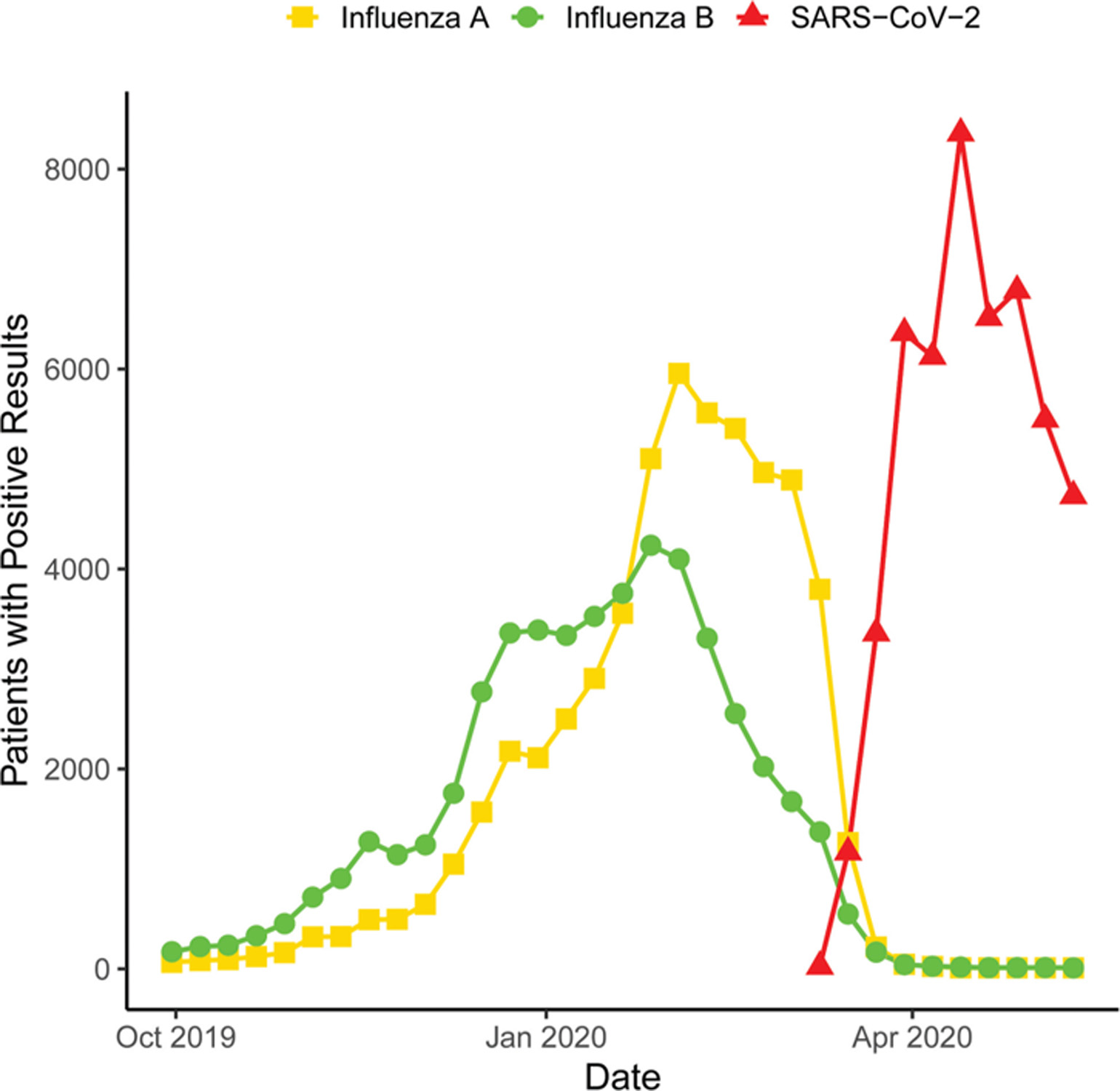
Counts of positive influenza A and B assays, as well as severe acute respiratory syndrome coronavirus-2 assays in Cosmos per week. The leveraged Logical Observation Identifiers Names and Codes are noted in Appendix A. As noted in the text, any counts under 10 (including 0) are obscured by rounding up to 10.

**Fig. 5:**
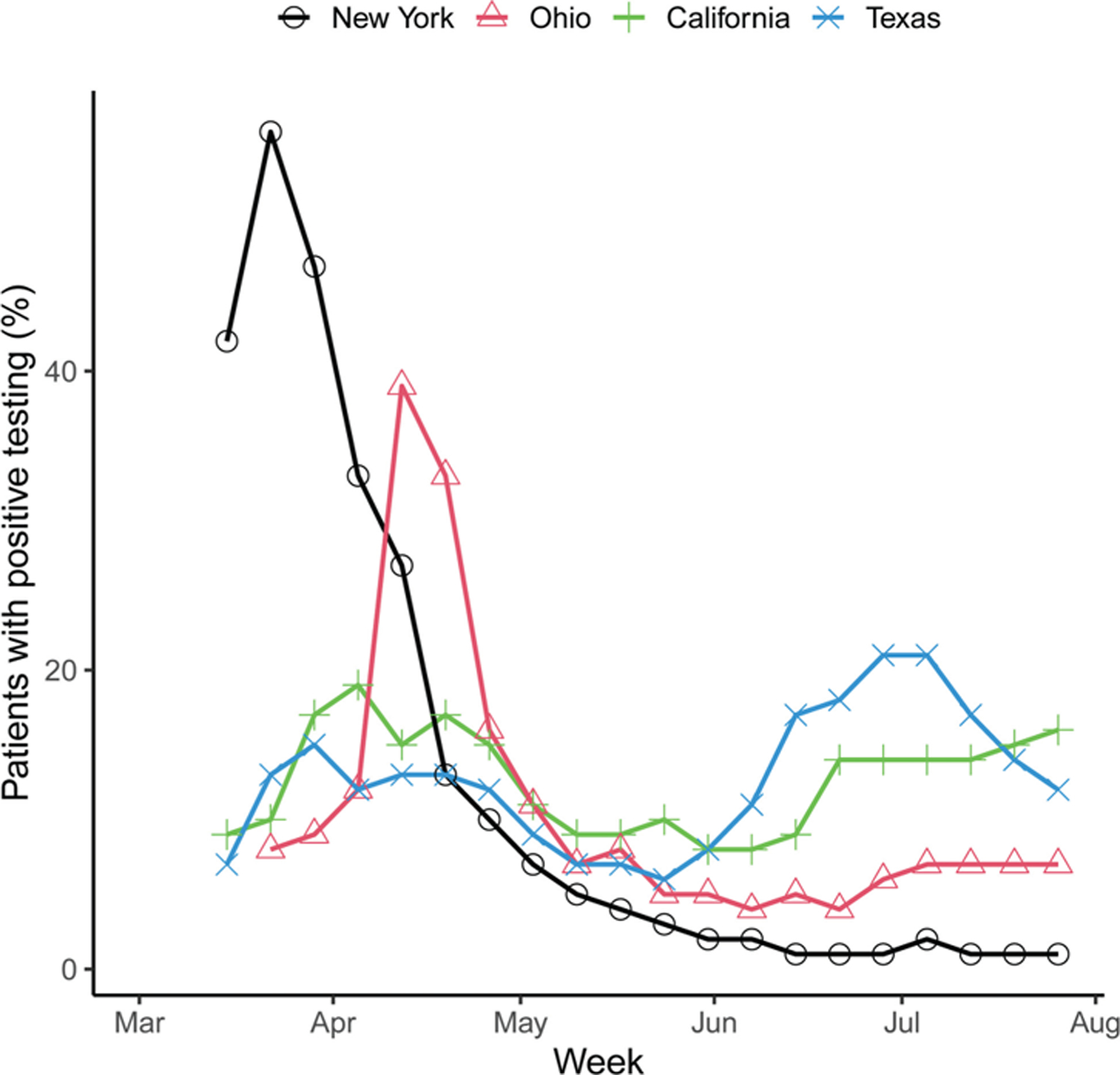
Weekly proportion of patients testing positive for severe acute respiratory syndrome coronavirus-2 in four selected states. The leveraged Logical Observation Identifiers Names and Codes are noted in Appendix A.

The CDC collects frequent data on positive influenza testing nationwide, providing critical epidemiologic information to public health and healthcare officials every season.^33^ A simple query of available laboratory data in Cosmos reveals distribution patterns of influenza A and B subtypes during the 2019 to 2020 flu season (Fig. 4). Adding positive testing for the severe acute respiratory syndrome coronavirus-2 (SARS-CoV-2) reveals the timing of this pandemic at the end of the typical flu season. Similar to CDC findings, Cosmos data revealed that the 2019 season began with an atypical preponderance of the influenza B subtype, later superseded by the A sub-type, and then a rapid rise in SARS-CoV-2 positivity (Fig. 4). As Cosmos collects patient zip code, the SARS-CoV-2 pandemic can be monitored both temporally and spatially at the national, state, county, and zip-code level. An assessment of the weekly number of patients testing positive for SARS-CoV-2 in four geographically distant states demonstrates the power of such an approach (Fig. 5).

### Immunization Utilization and Adherence Reporting: HPV Vaccination Adherence

While Cosmos can easily retrieve vaccination rates among different demographic cohorts, the availability of temporal inclusion operators can create more meaningful queries for vaccine series adherence (Table 2). For analysis on human papilloma virus (HPV) vaccination adherence, we queried initial and follow-up vaccination rates among adolescents with at least one outpatient visit. Our data revealed higher first dose adherence in 9- to 14-year-old Black patients compared with White patients (40.5 vs. 25.4%, *p* < 0.001), but poorer two-series vaccine completion rates in Black compared with White patients (40.5 vs. 57.5%, *p* < 0.001). These disparities are consistent with others’ findings.^34^

### Vaccine Adverse Event Reporting: MMRV and Febrile Seizures

The Vaccine Safety Datalink (VSD) is a valuable resource for vaccine related adverse event monitoring.^35^ Data from the VSD showed that children receiving the measles-mumps-rubella-varicella (MMRV) vaccine had a greater risk of febrile illness compared with measles-mumps-rubella with a separate varicella vaccine (MMR + varicella; 4.3 cases per 10,000 doses).^36^ Using Cosmos, we queried 11 to 23 months old who received either an MMRV (CVX 03) or MMR vaccine (CVX 94), in the past 10 years, then limited that cohort to patients who had an encounter or problem list diagnosis of febrile convulsion (SNOMED-CT 41497008) within 10 days of vaccination. In total, 55 of the 110,644 (0.049% or 5.0 per 10,000) receiving the MMRV had a febrile convulsion within 10 days, as opposed to 312 of the 1,041,705 (0.030% or 3.0 per 10,000) receiving the MMR. This represented a statistically significant excess risk of 2.0 per 10,000 doses (*p* < 0.001), in keeping with the published literature.^36^

### Health Services Research: Antibiotic Usage for Upper Respiratory Infections

The presence of encounter and medication prescription details in Cosmos allows for a wide range of queries to understand and quantitate the extent of important health services practices, such as potentially inappropriate use of antibiotics for upper respiratory infections (URI).^37,38^ In the emergency department (ED) setting specifically, antibiotic prescription frequency for URIs has decreased over time at the national level.^39^ We queried the number of patients per year who had an ED visit with an encounter diagnosis of URI (SNOMED-CT 54150009), and then stratified the result by age (<18 and 18) and any antibiotic prescription during that encounter. However, 2014 to 2017 estimates in adults and children fell within the confidence intervals of National Hospital Ambulatory Medical Care Survey (NHAMCS) data (Table 3).^39^ Though NHAMCS estimates were limited to data from 2017, we extended our query into 2019, revealing a continuing reduction in the percentage of patients receiving an antibiotic prescription frequency for URI over time in both adults (28.2 vs. 21.3, *p* < 0.001) and children (12.6 vs. 9.7, *p* < 0.001).

## Discussion

Cosmos is a rapidly growing EHR vendor-facilitated data collaboration that has accumulated data from over 60 million patients in under 2 years. The collaborative achieves a low barrier of entry for eligible healthcare facilities by leveraging existing clinical health information exchange infrastructure and data standards. Cosmos empowers customers with a user-friendly, flexible query building interface to generate new knowledge and insights.

Through several basic examples, we have shown how an intraplatform collaboration can potentially facilitate the evolution of institutions in their journey toward the goal of becoming learning healthcare systems. In mapping their data for submission to Cosmos, healthcare systems continue to standardize their data into common formats. Doing so allows for the meaningful aggregation of their data with other healthcare systems, and the ability to contrast their practices to those from other institutions. Such cross-institution data aggregation is a cornerstone of the success of other data collaboratives such as theNSQIPor the Mini-Sentinel initiative. In the case of NSQIP, the program lead to substantial improvements in surgical outcomes within the Veteran’s Association (VA) system, which lead to its rapid and international growth.^40^ With Mini-Sentinel, the volume of data needed to conduct certain analyses, such as assessing the risk of angioedema from different antihypertensive drugs, could only be realized with multi-institution aggregated health data.^41^ The current implementation of Cosmos is focused on data harmonization and aggregation, but the query and analytics tools are being rapidly evolved to promote similar initiatives.

The use cases presented serve to further broad initiatives such as chronic disease surveillance, syndromic surveillance, immunization adherence, and adverse event reporting and health services research. While many more advanced queries and research questions are possible, we elected to demonstrate simple queries with historical precedent as a proof of concept to focus our discussion on the collaboration itself. The queries presented can be modified or extrapolated to generate new insights necessary for learning healthcare systems to evolve. Chronic diseases surveillance can encompass myriad conditions beyond asthma or obesity. Vaccination adherence and adverse event monitoring can be extended to novel vaccines and vaccine formulations. Health disparities can be revealed and monitored, as demonstrated in our HPV vaccine example. Newoutbreaks, such as COVID-19, can be assessed nationwide. Finally, healthcare systems or even national organizations can implement practice changing education and processes that can be monitored at a national level, as suggested by antibiotic utilization in URIs example. Most of the use cases presented benefit from a greater power and generalizability achieved with larger datasets comprised of pooled data from different sources. The MMRV case use, for instance, measures a rare event that may not be easily captured a using one institution’s experience. The SARS-CoV-2 case also highlights interregional analyses that could not otherwise be done without multiregional collaboration.

Cosmos’ breadth of data types and filters allows for more nuanced cohort identification. Unlike claims-based systems such as Marketscan, Cosmos can leverage more than diagnoses for better disease phenotyping.^42^ This was broadly demonstrated in our obesity and asthma example, where reliance on diagnoses without measured BMI would greatly underestimate obesity. Many of the subject matter expert crafted “EHR phenotypes,” such as those from eMERGE consortium, could be reproduced by leveraging the multitude of data types and temporal parameters in Cosmos’ native query interface.^43^ Importantly, all Cosmos users have access to the same query building platform (called SlicerDicer) that they can use with their own identifiable data. This allows institutions to both generate and validate criteria-based phenotyping locally before adapting them to the larger, de-identified Cosmos dataset.

By design, Cosmos is an opt-in, all or none service for users of the same EHR platform. Since the initiative leverages preexisting technology infrastructures and health information exchange standards, customers need only agree to contribute data, and continue mapping their data items to established standards if they have not already done so. While several similar nationwide data aggregation collaborations exist, many require the involvement of separate third parties, and often, the need for an appliance behind a customer’s firewall.^9,11^ Such arrangements require new relationships, data-use agreements, and software or hardware implementations (with their associated costs) that may be a barrier to entry for some healthcare systems. Even regional networks such as the MDPHnet and NYC Macroscope require a great deal of external funding and multilevel collaborations that may not be available or accessible everywhere.^13,44–47^ To maintain accessibility and ensure Cosmos’ success, the Epic Corporation does not currently charge its customers any additional costs for participating in Cosmos.

A potential downside of an “all or none” collaboration from the contributing healthcare system’s perspective is concern regarding the loss of control over data contributed. Unlike other collaborations such as the Mini-Sentinel program or PCORnet, data contributions cannot be partial, and queries do not require approval at each site.^48^ To address this concern, contributions of a single patient or institution cannot be identified in Cosmos. This is done primarily by withholding line level data and limiting query results to >10 patients. Another potential concern for Cosmos contributors is the housing of an institution’s health information by the vendor in a central repository. To address this, the data transfer and storage processes are conducted securely and held to industry standards (the same standards used for health information exchange for clinical care). The Epic corporation owns and operates secure data centers under a comprehensive security program, which includes administrative, physical, and technical safeguards that follow industry best practices.^49^

Cosmos queries are only as strong as the available EHR data elements on which they are based. Data deduplication is not perfect, and data types are not harmonized across different Epic system. While the described mapping process may attenuate this heterogeneity, it is unlikely to remove it completely. In addition, true disease prevalence estimates in particular are notoriously difficult to ascertain from EHR data.^50^ In being limited to EHR data from a single vendor, Cosmos invariably comes with selection bias that can only partially be overcome by data accrual from tens of millions of patients.^15^ The non-randomly ascertained nature of the data and the bias associated with hospital systems that tend do use this specific EHR platform remain a major limitation for generalizability. The authors of this manuscript, from their perspective, strongly support cross-vendor collaboration and perceive within-vendor data harmonization as a requisite pre-step. Finally, the inability to re-sample or re-weight populations by demographics hinders data representation and generalizability.^51^

Cosmos provides a model for data standardization and aggregation that places the development and maintenance impetus on the EHR vendor, leveraging customers’ existing health information exchange networks and standards used for clinical health information exchange to minimize effort required to participate. Since Cosmos relies on existing clinical health informatics exchanges, C-CDAs, and generally accepted data standards, future versions of this endeavor could conceivably incorporate data from healthcare systems using different EHRs.

## Conclusion

Cosmos is a rapidly evolving resource that can assist institutions in their collective journeys toward becoming more complete learning healthcare systems. The initiative promotes within-vendor data standardization and aggregation, and presents a potential model for future customer driven inter-vendor EHR-based data collaborations.

## Supplementary Material

Supplementary material

Antibiotic RxNorm codes

## Figures and Tables

**Table 1:** Cosmos data variables as of June 2020

Concept	Discrete Data Variables
Demographics	Legal sex; gender identity; birth date; race; ethnicity; zip, county and state of patient; date of death; status of patient (alive or deceased); cause of death; gestational age at birth; language (spoken and preferred)
Encounter details	Start/end date and time; type, specialty; reason for visit; age at encounter; pregnancy status at encounter; place of service (zip, county and state); mode of arrival; discharge disposition; organization type
Problems	Diagnosis, including date noted and resolved
Diagnoses	Encounter based admission and discharge diagnoses; surgical diagnoses; visit (encounter) diagnoses; billing diagnoses
Surgical history	Procedure, date/time
Social history	Smoking status, duration and intensity; smoking start/stop dates; sexual activity, alcohol usage status; illegal drug usage status
Family history	Problem or pertinent negative; relationship to patient, age of onset, sex and status (living or deceased)
Outpatient medications	Medication name, type, dose, unit, route, frequency, dispense quantity, refills, and start/end date; indications of use
Allergies	Date noted; allergen; reaction; reaction severity; last updated instance
Immunizations	Immunization; administration date; route, dose; unit
Vital signs	Date/time; blood pressure; pulse; temperature; respiratory rate; oxygen saturation; height; weight; body mass index; head circumference.
Results	Procedure; date/time; specimen source; value and units; abnormal flag; reference range Microbiology organism, sensitivity and testing method if applicable
Procedure	Start/end date; procedure instant; billed procedure; provider specialty
Inpatient medications	Medication name, type, dose, unit, route, and start/end date
Birth data	APGAR score at 1, 5, and 10 min; nourishment method; delivery method; hospital days; birth count and order (if multiple)
Social determinants of health	Social connections; physical activity, stress; education; food insecurity, financial resource strain; intimate partner violence
Insurance	Medicaid, Medicare, privately insured or self-insured status

Note: Variables are grouped by concept.

Abbreviation: APGAR, appearance, pulse, grimace, activity, and respiration.

**Table 2:** Human papilloma virus vaccination and completion rates in patients between the ages of 9 and 14, stratified by race

Race	Total eligible^a^	HPV vaccine^b^ at least once	Patients vaccinated once who completed series within 12 mo
White	431,393	109,422 (25.4%)	62,918 (57.5%)
Black or African American	93,290	37,784 (40.5%)	15,302 (40.5%)
Asian	14,075	4,705 (33.4%)	2,521 (53.6%)
American Indian	3,063	898 (29.3%)	518 (57.7%)
Native Hawaiian	1,515	421 (27.9%)	230 (54.6%)

Abbreviation: HPV, Human papilloma virus.

a Eligibility was defined as any patient with a documented racial identity having at least one outpatient encounter between the ages of 9 and 14, between January 1, 2014 and December 31, 2019.

b Vaccination included bivalent HPV vaccination (CVX 118), quadrivalent HPV vaccination (CVX 62), 9-valent HPV vaccination (CVX 165), and “unspecified” HPV (CVX 137) vaccination.

**Table 3:** Number of patients seen at least once in an emergency room setting for a diagnosis of “upper respiratory infection,” with the number and percentage of those receiving an antibiotic during the encounter

	2010–2013	2014–2017	2018–2019
Age 18+			
All patients with ED visit for URI	18,881	95,598	179,140
Number of patients with ED visits for URI with antibiotic ordering (%, 95% confidence interval)	8,694 (46.1%; 45.3–46.8)	27,001 (28.2%; 28.0–28.5)^a^	38,327 (21.3%; 21.2–21.6)
Age < 18			
All patients with ED visit for URI	70,499	244,563	333,714
Number of patients with ED visits for URI with antibiotic ordering (%, 95% confidence interval)	13,377 (19.0%; 18.7–19.3)	30,826 (12.6%; 12.5–12.7)^b^	32,482 (9.7%; 9.7–9.9)

Abbreviations: CI, confidence interval; ED, emergency department; NMHACS, National Hospital Ambulatory Medical Care Survey; URI, upper respiratory tract infection.

a Comparable estimates based on NHAMCS data are 32.0 (95% CI: 22.0–43.5).

b Comparable estimates based on NHAMCS data are 10.1 (95% CI: 7.4–13.9).

Note: Antibiotic usage was based on 1,861 RxNorm codes that code for antibiotics (noted in supplementary material).

## Clinical Relevance Statement

Seamless data aggregation and population-level analytics between healthcare systems may be an important step in the evolution of all learning healthcare systems. A cross-institution, vendor-facilitated and health information exchange dependent data aggregation and analysis platform shows promise in facilitating this goal among users of the same EHR. Our experience shows the power and scalability of such an “all or none” contribution model, while highlighting numerous concerns that may be associated with such an approach.

## Appendix

**Appendix A:** Logical Observation Identifiers Names and Codes

LOINC codes for influenza A	LOINC codes for influenza B	LOINC codes for SARS-CoV-2
44564–3	46083–2	94500–6
46082–4	44573–4	94314–2
44561–9	44574–2	94309–2
44558–5	80383–3	94306–8
80382–5	44572–6	94534–5
44559–3	5867–7	41458–1
5863–6	76080–1	41459–9
76078–5	82170–2	94531–1
48310–7	38382–8	
82166–0	40982–1	
31858–4	44575–9	
44563–5	44577–5	
43874–7	43895–2	
31859–2	31864–2	
5864–4	49534–1	
44560–1	5866–9	
5861–0	92976–0	
5862–8	85478–6	
49531–7	5865–1	
38381–0		
34487–9		
92977–8		
85477–8		
22827–0		
40891–3		

Abbreviations: LOINC, Logical Observation Identifiers Names and Codes; SARS-CoV-2, severe acute respiratory syndrome coronavirus-2.
