# Supplementary material for: The Cosmos Collaborative: A Vendor-Facilitated Electronic Health Record Data Aggregation Platform
Source: ACI open. Author manuscript; Available in PMC 2022 Jan 20. (PMC8775787; doi:10.1055/s-0041-1731004)
Supplement: Supplementary material [file NIHMS1762373-supplement-Supplementary_material.docx]

## Supplementary material:

Supplementary Table 1:

| LOINC codes for influenza A: | LOINC codes for influenza B: | LOINC codes for SARS-CoV-2: |
| --- | --- | --- |
| 44564-3 | 46083-2 | 94500-6 |
| 46082-4 | 44573-4 | 94314-2 |
| 44561-9 | 44574-2 | 94309-2 |
| 44558-5 | 80383-3 | 94306-8 |
| 80382-5 | 44572-6 | 94534-5 |
| 44559-3 | 5867-7 | 41458-1 |
| 5863-6 | 76080-1 | 41459-9 |
| 76078-5 | 82170-2 | 94531-1 |
| 48310-7 | 38382-8 |  |
| 82166-0 | 40982-1 |  |
| 31858-4 | 44575-9 |  |
| 44563-5 | 44577-5 |  |
| 43874-7 | 43895-2 |  |
| 31859-2 | 31864-2 |  |
| 5864-4 | 49534-1 |  |
| 44560-1 | 5866-9 |  |
| 5861-0 | 92976-0 |  |
| 5862-8 | 85478-6 |  |
| 49531-7 | 5865-1 |  |
| 38381-0 |  |  |
| 34487-9 |  |  |
| 92977-8 |  |  |
| 85477-8 |  |  |
| 22827-0 |  |  |
| 40891-3 |  |  |
